# An analysis on HBsAg, Anti-HCV, Anti-HIV½ and VDRL test results in blood donors according to gender, age range and years

**DOI:** 10.1371/journal.pone.0219709

**Published:** 2019-09-19

**Authors:** Canan Eren

**Affiliations:** Marmara University Pendik Training and Research Hospital, Medical Microbiology and Blood Centre, Pendik, Istanbul; Duke University, UNITED STATES

## Abstract

**Objective:**

Blood transfusion is the most frequently used and life-saving therapeutic procedure today. Transmission of virus, bacteria and parasitic microorganisms may occur due to transfusion (Transfusion transmitted infections-TTIs). Hepatitis B and C, human immunodeficiency virus (HIV) and syphilis (*Treponema pallidum*) bear the risk of transmission by transfusion. Hepatitis B surface antigen (HBsAg), anti-HCV, anti-HIV½ and syphilis antibody (VDRL: Venereal Disease Research Laboratory) are routinely controlled in all donated blood samples. The aim of the present study was to analyze the seroprevalence rates of blood donors through screening test results according to duration, age range and gender.

**Material and methods:**

Data of all blood donors obtained from blood Centre of Marmara University Pendik Training and Research Hospital between January 2013 and October 2018 were analyzed retrospectively. Serum samples of the donors were analyzed for HBsAg, anti-HCV, anti-HIV½ and VDRL. Test results of 114.240 donors were scanned. Gender, age range and distribution by years of these donors were analyzed. According to age distribution of donors were divided into 4 groups.

**Results:**

There were 114.240 participants including 106.153 (92.9%) males and 8.087 (7.1%) females. The positivity rate of HBsAg was detected 0.4% (36/8087) in females and 0.5% (500/106.153) in males. The positivity rate of anti-HCV was detected 0.4% (32/8.087) in females and 0.3% (344/106.153) in males. The positivity rate of anti HIV½ was 0.1% (9/8.087) in females and 0.1% (92/106.153) in males whereas the positivity rate of VDRL was 0.5% (41/8.087) in females and 0.3% (362/ 106.153) in males. Positivity rate for HBsAg and HCV were lower in the cases between 18 and 30 years of age. The positivity rates for anti-HIV½ was not significantly different according to the age range. Positivity rate for VDRL was higher in the cases at 51 years of age and older.

**Conclusion:**

No difference was found between men and women in terms of HBsAg, anti-HCV and anti-HIV½positivity. However, VDRL test positivity was significantly higher in female participants. Furthermore, HBsAg, anti-HCV and VDRL positivity rates increased by age.

## Introduction

Blood transfusion is the most commonly used and life-saving therapeutic procedure. However, some infectious complications may develop during this procedure. Transmission of virus, bacteria and parasitic microorganisms may occur due to transfusion (Transfusion transmitted infections- TTIs). Hepatitis B and C, HIV and syphilis (*T*. *Pallidum*) are important infections. In order to minimize this risk, it is important to be careful about donor selection criteria and conduction of screening tests against these 4 infectious agents in a donor blood [1–3].

The World Health Organization (WHO) recommends that all blood donations should be screened for selected infections prior to use and that screening should be mandatory for HBV, HCV, HIV and *T*. *Pallidum* [4]. As a part of national blood banking procedures, HBsAg, anti-HCV, anti-HIV½ and VDRL are analyzed in all donated blood samples [5]. Despite performance of these tests, transmission of viral infections is still hazardous in the early stage [window period] where the serological markers are negative [6,7].

HBsAg positivity in healthy individuals ranged between 2.4% and 9% and anti-HCV positivity ranged from 1% to 2.2% in our country depending on regions and time period [8,9]. The 2015 global prevalence of HBV infection in general population reported by WHO was 3.5% for about 257 million people. Global prevalence of HCV reported by WHO is 71 million people with HCV infection in the world accounting for 1% of the population [10].

It is estimated that 0.8% of adults between 15 and 45 years of age live with HIV globally. However, Sub-Saharan Africa is the most severely affected territory for HIV with almost 1 individual per 25 adults [4.2%] [11]. In our country, there are 6.188 HIV positive cases according to 2012 data of Ministry of Health [8].

The global incidence of syphilis was 25.1 cases per 100.000 adults among 55 countries that were reported in the GAVDRL (Global Aids Response Progress Reports) in 2014 [12]. In our country, these rates range between 0.001% and 0.13% in blood donors [6].

Our blood centre is one of the largest blood centers in Istanbul. Since Istanbul is exposed to internal migration from all regions of Turkey, the data may reflect the whole country. The aim of the present study was to analyze the results of screening tests of blood donors who referred to our blood centre through seroprevalence rates according to gender, age range and duration, and to make a contribution to the literature by comparing the results of our country and the world.

## Materials and methods

Data of all blood donors obtained from blood Centre of Marmara University Pendik Training and Research Hospital between January 2013 and October 2018 were analyzed retrospectively.

The candidates for blood donation completed the donor inquiry form and a physical examination was performed by blood centre doctor. Subsequently, serum samples of the candidates who were eligible to be donors were analyzed for HBsAg, anti-HCV, anti-HIV½ and VDRL. The study included healthy male and female individuals between 18 and 65 years of age with a body weight of >50 kg. Minimum hemoglobin levels were accepted as 12.5 g/dl in females and 13.5 g/dl in males. Test results of 114.240 donors were scanned. The gender, age range and distribution by years of these donors were analyzed. The donors were divided into 4 groups according to the age range; 18–30 years, 31–40 years, 41–50 years and over 51 years. HBsAg, anti-HCV and anti-HIV½ tests were performed on serum samples obtained from donors through microparticle- enzyme immunoassay (EIA) (Abbott Architect i2000 SR Combo diagnostic kits, USA) method. VDRL test was used for syphilis screening.

Samples with positive test results were re-tested by same method; same equipments and same serum samples were utilized for repetitive reactivity. Repetitive reagent samples were considered positive. Seropositive samples for HIV½ were referred to Infectious Diseases department of Istanbul provincial health directorate for Western Blot (WB) validation test.

### Statistical analysis

Statistical analysis was conducted by NCSS (Number Cruncher Statistical System) 2007 Statistical Software (Utah, USA) program. In addition to descriptive statistical methods and determination of the prevalence of serological tests, Bootstrap (1000 samples) within a confidence interval by 95% was used. Pearson Chi-Square tests and Fisher’s Exact test were used for comparison of qualitative data. The results were evaluated within 95% confidence interval and at a significance level of p<0.05.

### Statement of ethics

The present study was approved by Ethics Committee for Clinical Researches of Faculty of Medicine within Marmara University by decision number of 09.2018.358.

## Results

Total number of participants was 114.240 including 106.153 (92.9%) males and 8.087 (7.1%) females. The age average of the males and females according to positive tests is presented in Table 1.

**Table 1:** Age average of positive HBsAg, anti-HCV, anti-HIV½ and VDRL results by gender.

Seropositive test	Average age of males	Average age of females	*Average age*
**HbsAg**	37.09±9.77 *(95%CI 36*.*41–43*.*48)*	39.94±10.45 *(95%CI 36*.*24–37*.*91)*	37.26±9.83 *(95%CI 36*.*45–38*.*07)*
**anti-HCV**	35.08±9.63 *(95%CI 34*.*81–36*.*79)*	35.91±9.92 *(95%CI 32*.*45–39*.*37)*	35.81±9.64 *(95%CI 34*.*86–36*.*76)*
**anti-HIV**½	35.55±9.57 *(95%CI 33*.*61–35*.*51)*	29.33±8.63 *(95%CI 22*.*69–35*.*96)*	35.19±9.61 *(95%CI 33*.*15–36*.*89)*
**VDRL**	38.14±10.26 *(95%CI 37*.*15–39*.*14)*	37.13±10.76 *(95%CI 33*.*93–40*.*33)*	38.04±10.31 *(95%CI 37*.*09–38*.*99)*

When all positivity rates of donors were reviewed in total; one test was detected positive in 118 (0.36%) females and 1.298 (0.31%) males (Table 2).

Five hundred and thirty six HBsAg-positive samples included 500 (93.3%) males and 36 (6.7%) females. Total HBsAg positivity rate was 0.5% (536/114.240). The sero-positivity rate of HBsAg was detected 0.4% (36/8087) in females and 0.5% (500/106.153) in males.

Three hundred and seventy six anti-HCV positive analyses included 344 (91.5%) males and 32 (8.5%) females. Total Anti-HCV positivity was 0.3% (37/114.240). The positivity rate was detected 0.4% (32/8.087) in females and 0.3% (344/106.153) in males.

There were101 samples with positive anti-HIV½ analysis including 92 (91.1%) males and 9 (8.5%) females. The rate of positive anti HIV½ antigens was found 0.1% (101/114.240) in total; the positivity rate was 0.1% (9/8.087) in females and 0.1% (92/106.153) in males.

There were 403 samples with positive VDRL test;362 (89.8%) were male and 41 (10.2%) were female. Total rate of positive VDRL test was found 0.4% (403/114,240); the positivity rate was 0.5% (41/8.087) in females and 0.3% (362/106.153) in males (Tables 2 and 3).

**Table 2:** Distribution of positivity rates by gender.

Positive test		F			M	
		[n = 8087]			[n = 106.153]	
	n	%	95% CI	n	%	95% CI
**HbsAg**	36	0.4	0.31–0.59	500	0.5	0.43–0.51
**anti-HCV**	32	0.4	0.26–0.54	344	0.3	0.29–0.36
**anti-HIV½**	9	0.1	0.05–0.20	92	0.1	0.07–0.10
**VDRL**	41	0.5	0.36–0.66	362	0.3	0.31–0.38
**Total**	**118**	**0.36**	**0.30–0.43**	**1298**	**0.31**	**0.29–0.32**

F:Female, M:Male 95% CI: 95% Confidence Interval Bootstrap for Percent: (1000 Samples)

**Table 3:** Analysis of positivity rates of HBsAg, anti-HCV, anti-HIV½ and VDRL according to age range and gender.

		HbsAg	anti-HCV	anti-HIV½	VDRL
		(+)	(+)	(+)	(+)
**Total**	**N**	**536**	**376**	**101**	**403**
	**%**	0.47	0.33	0.09	0.35
	**95% CI**	0.43–0.51	0.30–0.37	0.07–0.11	0.32–0.39
**Female** *(n = 8087)*	**N**	36	32	9	41
	**%**	0.45	0.40	0.11	0.51
	**95% CI**	0.31–0.59	0.26–0.54	0.05–0.2	0.36–0.66
**Male** *(n = 106153)*	**N**	500	344	92	362
	**%**	0.47	0.32	0.09	0.34
	**95% CI**	0.43–0.51	0.29–0.36	0.07–0.1	0.31–0.38
	**P**^**a**^	0.743	0.278	0.473	0.015*
^**A**^**18-30 years** *(n = 44200)*	**N**	138	122	35	117
	**%**	0.31	0.28	0.08	0.26
	**95% CI**	0.26–0.36	0.23–0.33	0.05–0.11	0.22–0.31
^**B**^**31-40 years** *(n = 41136)*	**N**	185	144	30	116
	**%**	0.45	0.35	0.07	0.28
	**95% CI**	0.39–0.52	0.29–0.41	0.05–0.1	0.23–0.33
^**C**^**41-50 years** *(n = 22231)*	**N**	161	78	30	109
	**%**	0.72	0.35	0.13	0.49
	**95% CI**	0.62–0.84	0.27–0.43	0.09–0.19	0.4–0.59
^**D**^**≥ 51 years** *(n = 6673)*	**N**	52	32	4	61
	**%**	0.78	0.48	0.06	0.91
	**95% CI**	0.56–0.99	0.32–0.66	0.01–0.13	0.7–1.15
	**P**^**a**^	0.001**	0.063	0.102	0.001**
		A>C, D			D>A,B

95% CI: *95% Confidence Interval Bootstrap for Percent (1000 samples)*

^a^Pearson’s Chi-square Test

*p<0.05

**p<0.01

Prevalence values of HBsAg, anti-HCV, anti-HIV½ and VDRL as well as associated Confidence Interval (CI) by 95% in serological tests of 114.240 donors are presented in Table 3.

Total prevalence was detected as follows; 0.47% *(95% CI*: *0*.*43–0*.*51*) for HBsAg; 0.33% *(95% CI*:*0*.*30–0*.*37*) for anti-HCV; 0.09% *(95% CI*: *0*.*07–0*.*11*) for anti-HIV½ and %0.35 *(95%CI*: *0*.*32–0*.*39*) for VDRL. Prevalence and 95% CI values according to gender and age are presented in Table 3.

When age range of the donors are reviewed, majority of the donors are grouped between 18 and 30 years of age (38.7%). The age group of 51 years and older was the least age group with positive results by 5.6% (Table 3).

Positivity rate for HBsAg was significantly lower in the cases between 18 and 30 years of age when compared with the cases between 41 and 50 years of age as well as the cases at 51 years of age and older. The HBsAg rates increase by the increase in age.

A lower prevalence rate was detected for anti-HCV in the cases between 18 and 30 years of age when compared with the cases at 51 years of age and older. Overall review indicated that anti-HCV rates increased by age.

The positivity rates for anti-HIV½ were not significantly different according to the age range.

**Fig 1:**
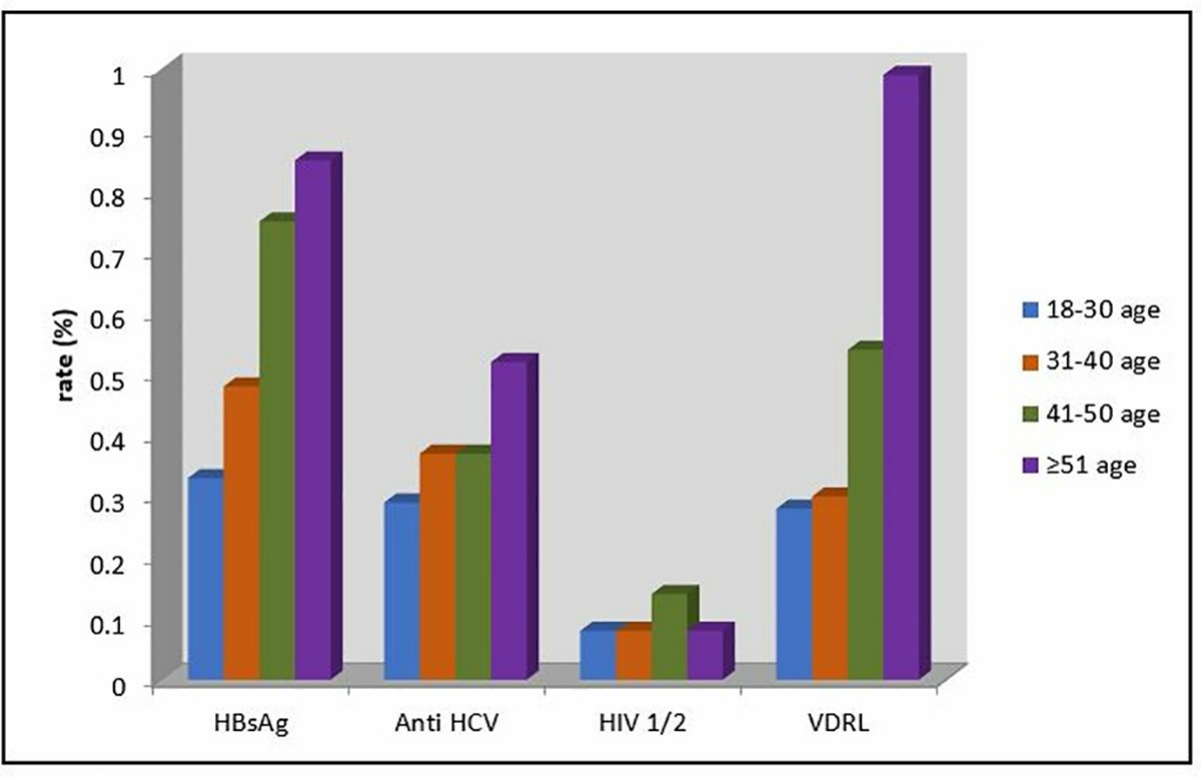
Positivity rates of serological tests depending on the age ranges.

**Fig 2:**
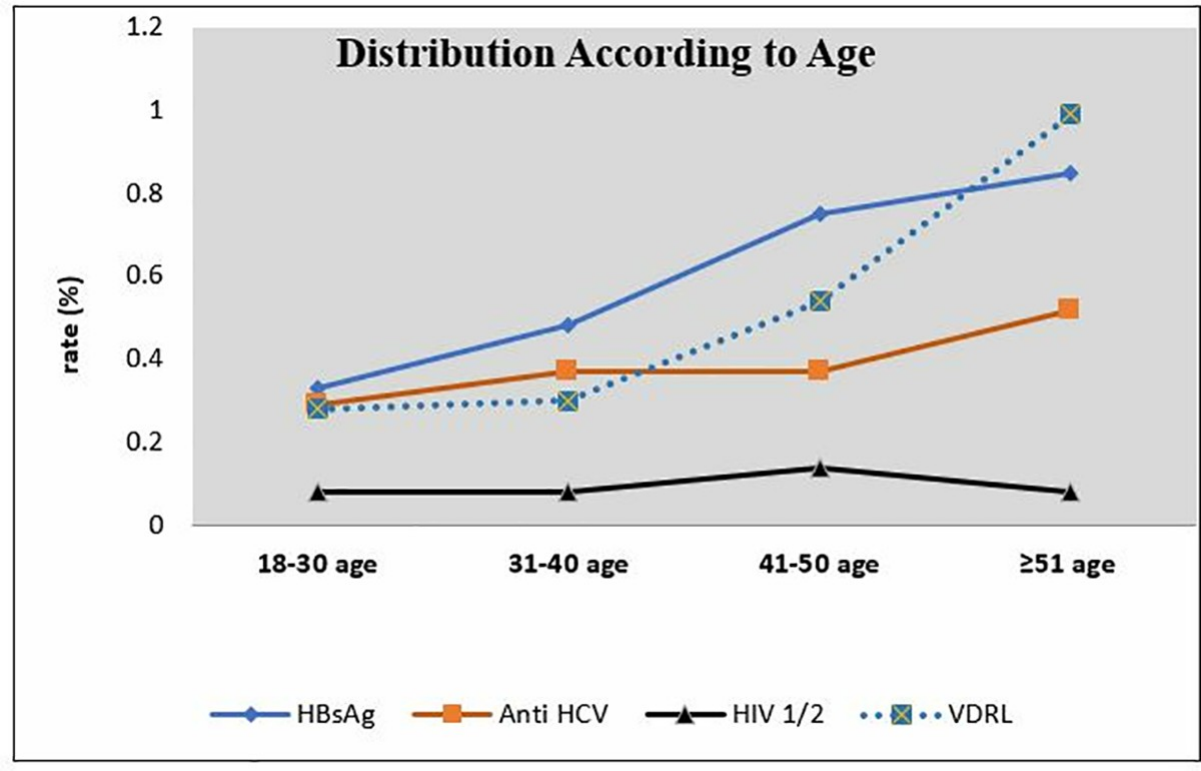
Sero-positive rates according to age groups.

Positivity rate for VDRL was significantly higher in the cases at 51 years of age and older when compared with the cases between 18 and 50 years of age as well as the cases between 31 and 40 years of age. The VDRL rates also increase by the increase of age. The rates of positive tests according to age ranges are presented in Figs 1 and 2.

**Fig 3:**
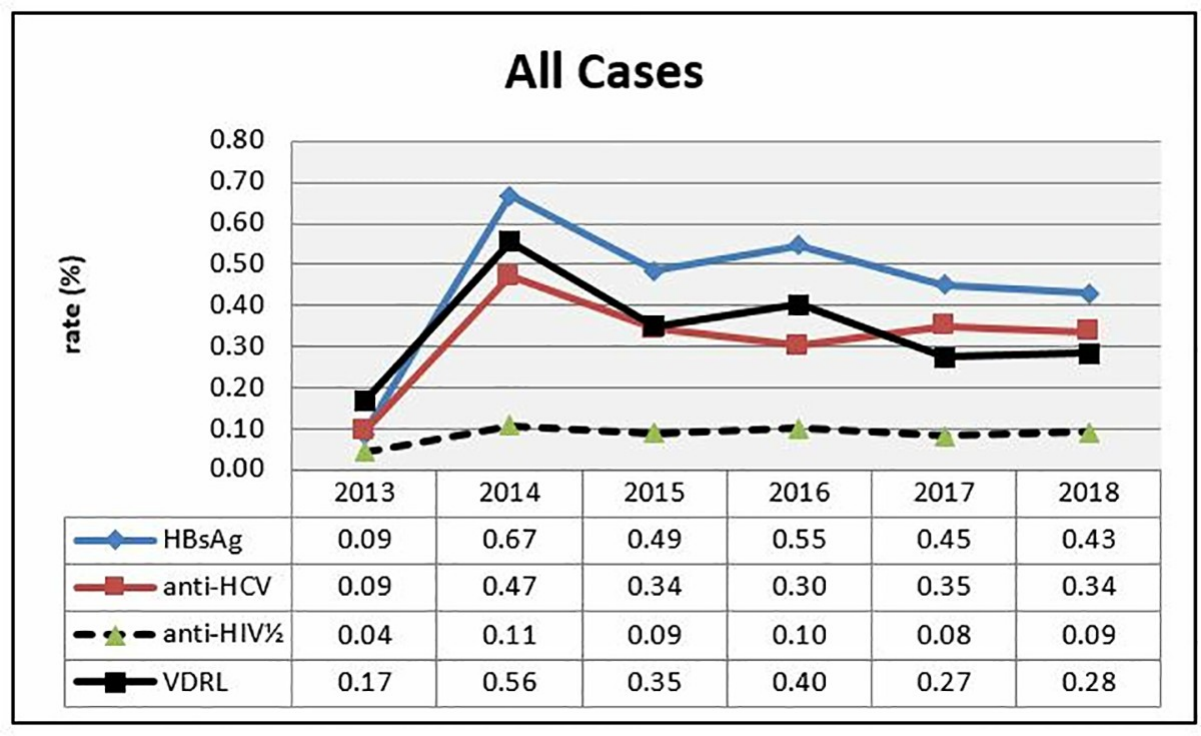
Distribution of positive test rates by years.

The prevalence values for HBsAg, anti-HCV, anti-HIV½ and VDRL between 2013 and 2018 were shown in the table and figure (Table 4) (Fig 3).

**Table 4:** The distribution analysis of positivity rates for HBsAg, anti-HCV, anti-HIV½ and VDRL according to the gender and years.

Year		Total				Female			Male			p
		N		%	95% CI	N	%	95% CI	n	%	95% CI	
	**HbsAg**											
**2013**	**(+)**		12	0.09	0.04–0.14	1	0.12	0–0.38	11	0.08	0.04–0.13	^b^0.532
**2014**	**(+)**		142	0.67	0.56–0.78	8	0.6	0.24–1.05	134	0.67	0.56–0.78	0.774
**2015**	**(+)**		103	0.49	0.4–0.58	6	0.43	0.14–0.83	97	0.49	0.4–0.59	0.737
**2016**	**(+)**		120	0.55	0.46–0.65	5	0.3	0.06–0.62	115	0.57	0.46–0.68	0.161
**2017**	**(+)**		94	0.45	0.36–0.54	10	0.61	0.31–1.04	84	0.44	0.35–0.54	0.316
**2018**	**(+)**		65	0.43	0.32–0.54	6	0.5	0.17–0.92	59	0.42	0.32–0.53	0.694
	**anti-HCV**											
**2013**	**(+)**	13		0.09	0.05–0.14	3	0.35	0–0.83	10	0.08	0.03–0.13	^b^0.041*
**2014**	**(+)**	100		0.47	0.38–0.56	7	0.52	0.22–0.97	93	0.47	0.38–0.56	0.771
**2015**	**(+)**	73		0.34	0.27–0.42	4	0.28	0.07–0.6	69	0.35	0.27–0.43	^b^1.000
**2016**	**(+)**	66		0.30	0.23–0.38	5	0.3	0.06–0.62	61	0.3	0.23–0.38	^b^1.000
**2017**	**(+)**	73		0.35	0.27–0.44	10	0.61	0.24–1.01	63	0.33	0.25–0.42	0.063
**2018**	**(+)**	51		0.34	0.24–0.,44	3	0.25	0–0.58	48	0.34	0.25–0.45	^b^0.796
	**anti-HIV½**											
**2013**	**(+)**	6		0.04	0.01–0.09	1	0.12	0–0.38	5	0.04	0.01–0.08	^b^0.316
**2014**	**(+)**	23		0.11	0.07–0.16	5	0.37	0.08–0.75	18	0.09	0.05–0.14	^b^0.013*
**2015**	**(+)**	19		0.09	0.05–0.13	0	0	-	19	0.1	0.05–0.14	^b^0.634
**2016**	**(+)**	22		0.10	0.06–0.14	0	0	-	22	0.11	0.07–0.15	^b^0.406
**2017**	**(+)**	17		0.08	0.04–0.13	2	0.12	0–0.31	15	0.08	0.04–0.12	^b^0.391
**2018**	**(+)**	14		0.09	0.05–0.15	1	0.08	0–0.27	13	0.09	0.04–0.15	^b^1.000
	**VDRL**											
**2013**	**(+)**	23		0.17	0.1–0.23	2	0.24	0–0.63	21	0.16	0.09–0.23	^b^0.649
**2014**	**(+)**	118		0.56	0.46–0.66	14	1.05	0.56–1.64	104	0.52	0.42–0.62	0.013*
**2015**	**(+)**	74		0.35	0.27–0.43	8	0.57	0.21–1.06	66	0.33	0.25–0.41	^b^0.136
**2016**	**(+)**	88		0.40	0.32–0.49	9	0.54	0.19–0.91	79	0.39	0.3–0.48	^b^0.337
**2017**	**(+)**	57		0.27	0.21–0.34	3	0.18	0–0.43	54	0.28	0.2–0.36	^b^0.623
**2018**	**(+)**	43		0.28	0.21–0.38	5	0.42	0.09–0.83	38	0.27	0.19–0.37	^b^0.387

Pearson’s Chi-square Test

^b^Fisher’s Exact Test

*p<0.05

There was not any significant difference between positivity rates for HBsAg; Anti HCV and HIV½ depending on the gender (p>0.05); VDRL positivity was found significantly higher in female cases when compared with the males (p<0.05).

A statistically significant difference was detected between HBsAg positivity rates depending on the age ranges (p<0.01); positivity rates for HBsAg was significantly higher in the age range of 18–30 years than age range of 41–50 and 51–60 years. No difference was found between anti-HCV and HIV½ rates depending on the age (p>0.05). A statistically significant difference was detected in VDRL positivity rates according to the age (p<0.01); VDRL positivity rates were found significantly higher in the age range of 51–60 years than the age ranges of 18–30 and 31–40 years.

There was not any statistically significant difference detected in positivity rates of HBsAg, HIV½ and VDRL depending on the gender in 2013 (p>0.05); however, anti-HCV was significantly higher in female cases than males (p<0.05).

No statistically significant difference was detected in HBsAg and anti-HCV positivity rates depending on the gender in 2014 (p>0.05); however, anti-HIV½ and VDRL were significantly higher in the female cases than males (p<0.05).

There was not any significant difference detected in HBsAg, anti-HCV, anti HIV ½ and VDRL positivity rates depending on genders in years 2015, 2016, 2017 and 2018 (p>0.05).

## Discussion

Providing a safe blood transfusion is essential, this is also the primary target of blood centers. Detection of infectious diseases through transfusion in the blood of candidates for blood donation would present a possible infection to the receiver. Countries determine whether an agent should be screened before transfusion according to prevalence of carrier states in their populations. In our country, screening tests for HBsAg, anti-HCV, anti-HIV½ and syphilis are performed [13].

Majority of our donor candidates consisted of males (92.9%). The rate of female donors was 7.1%. This result is comparable with other studies of different countries and our country. In general, number of male donors is much more than females in Africa, Asian and Middle East countries [6, 8, 14–16]. Such male dominance may be explained by the belief that males are healthier than females and they are more suitable for blood donation. In addition, females are believed that they have blood donations naturally through their menstrual cycle every month. Other obstetrical factors including pregnancy and breastfeeding also restrict many females from donating blood [14,15]. However, female and male donor rates are close to each other in United States of America and Western European countries [16]. In the study of Cao et al. from China, rate of female donor was more than males by 50.6% [17].

Incidence of post-transfusion hepatitis is 2/10.000 for each recipient of blood and blood products. Rate of HBV infection is 0.3–1.7% in post transfusion hepatitis [13]. Infectious agents which spread by transfusion are detected in blood donor candidates all over the world and differences are detected between the countries and regions. HBsAg positivity rates in blood donors are found 0.06% in USA [18], 0.7–2.2% in India [19], and 0.2% in Mexico. Such rate is between 0.4% and 4.2% in our country. However, prevalence of HBsAg positivity was significantly higher in Sub-Saharan African countries and China. The rates in aforementioned countries are between 4.1% and 18.6% in Nigeria [16], 4.7% in Ethiopia [20], 8.8% in Tanzania, 13.9% in Mali [21], 10.6% in Mozambique [19], 10.1% in Cameroon, and between 7.18% and 10.9% in China [17]. Our data belonged to Istanbul province and our rate for positive HBsAg was 0.5%. The rate in female blood donor candidates was 0.4% whereas positivity rate was 0.5% in male candidates in the present study. There was not any significant difference detected between female and male positivity rates.

Our rates were at the lowest rate level in our country. Positivity rates differ in different regions of the country. There is a tendency of decrease of HBV infections in the Western region when compared with the Eastern regions [6]. Our rates tend to decrease since 2014. In consideration of such decrease, the causes that reduce the prevalence may include awareness of the population about HBV infection, health precautions, increase of vaccination, revision of donor inquiry form, careful donor examination and exclusion of the individuals with suspicious of any infectious disease at preliminary stage. Furthermore, periodical trainings that blood bank staff is exposed about blood banking and transfusion medicine may be effective in such decrease.

We divided donor candidates into 4 different age groups. Such age groups were 18–30, 31–40, 41–50 and above 50 years. HBsAg positivity was the least in 18–30 years age group. The positivity rate at that age range [0.26%] was significantly lower than other age groups. Turan et al. from Turkey detected no difference in HBsAg detection probability between the age groups [22]; however, Noubiap et al. from Cameron found that HBsAg detection probability was higher in younger age groups [14]. This was associated with insufficient vaccination and protection programs. In the study of Kader et al. from Turkey, the average for HBsAg positivity was 31.3 [5]. Niazi et al. from Pakistan found positivity rate in the 18–30 age group similar to our study [23]. An effective vaccination program in our region probably reduced HBsAg positivity rates in our young age group in the last decade.

The 2015 global prevalence of HCV reported by WHO was 71 million people with HCV infection in the world, accounting for 1% of the population [10,16]. The actual agent for hepatitis spread by transfusion is HCV. Cirrhosis and hepatocellular carcinoma develops at least in 25% of chronic cases. A review of anti-HCV positivity rates indicates that the rates are higher in Sub-Saharan African countries. Noubiap et al. from Cameron found such rate 4.8% [14] whereas Fiekumo et al. [19] and Okoroiwu et al. from Nigeria found the rates 6% and 3.6%, respectively. The rates are significantly higher in outcomes of the studies carried out in Tanzania, Nigeria and Mali[16, 20, 21]. Positivity rate in different regions of India vary between 0.23% and 1.02% [19]; the positivity rate in China [17] and European region [10] were 1.43% and 1.5%, respectively.

Anti-HCV positivity rate in our country was found between 0.16% and 0.6% in different regions of the country [5, 6, 8, 13, 22, 24]. Anti-HCV positivity rate was detected 0.3% in the present study. This was consistent with other rates detected in our country. The rate for anti-HCV positivity was 0.4% in females and 0.3% in males; and there was not any significant difference in the present study. In consideration of age range groups, there was not any statistically significant difference detected between anti-HCV positivity rates. Kader et al. from Turkey did not find any difference between positivity rates in terms of gender [5]. Cao et al. also did not find any significant difference for anti-HCV positivity between genders [17].

In our study, there was not any significant difference according to the age groups in review of anti-HCV positivity in terms of age groups. Such findings obtained according to the gender and age groups provided similar results with the study conducted by Agus et al. from Turkey [5]. Cao et al. found anti-HCV positivity rate higher in 41–70 age range in their study [1.76%]. In China, Blood Donation Law which focuses on blood transfusion as an important transmission route for HCV and HIV came into effect in 1998 [17]. Niazi et al. from Pakistan found anti-HCV positivity rate as significant between 31 and 45 years of age [23].

Global prevalence of HIV which mostly spread through blood transfusion and sexual intercourse is 1.1% in adults. Globally, 36.7 million people live with HIV at the end of 2016. The burden of the epidemia continues between countries and regions. Sub-Saharan Africa is the most affected country with almost 1 patient per 25 adults (4.2%) living with HIV, which accounts for nearly two-third of people living with HIV worldwide [11, 16]. Positivity rates in Mali, Ethiopia and Tanzania are 2.6%, 3.1%, 3.8% and 3.8%, respectively. Another study from Nigeria detected such rate as 10% [25]. Positive anti-HIV rates vary between 0.26% and 0.56% in India [19].

The blood donors with anti-HIV positivity vary between 0% and 0.2% in our country [8]. Anti-HIV positivity rate was found 0.1% in the present study. This is similar with other rates detected in our country. There was not any significant difference between anti-HIV positivity rates according to the genders in our study. Such rate was found as 0.16% in the study conducted by Cao et al. in China. Whereas a significant difference was found between females and males (0.10% vs. 0.23%)[17].

There was not any significant difference between anti-HIV rates in terms of age ranges. In the study of Cao et al., the positivity rate was found significantly higher in the age range between 21 and 30 years. It is reported that such age group is sexually active. Drug use and high-risk sexual behaviors exist in the regions with higher HIV infection rate [17]. In accordance with our work, there was not any significant difference for anti-HIV positivity in terms of gender and age range in the study of Agus et al. from our country [26].

The donor samples with repetitive reactivity for HIV were referred to infectious diseases office of Provincial Health Directorate of Istanbul for confirmation tests; and confirmation tests were run by Western-Blot method. Only two samples were detected positive among the samples referred for confirmation test. Higher rates of false positive results in patients may be associated with the drugs or cross reaction of antibodies with HIV antibodies because of their diseases.

Post-transfusion syphilis is a rare condition which is easily treated after diagnosis. The spread risk of syphilis as a result of blood and blood product transfusion is lower since the agent loses its infectivity in the blood in the refrigerator after 72 hours. It is not one of the compulsory screening tests in some countries. However, screening tests are performed in many countries, because its presence can be a marker of donor’s life style [13].

There are different rates of positive VDRL test ranging between 0.001% and 2.33% among blood donors in our country. The variation in the rates appears due to geographical differences, methodological differences, differences of kits, and confirmations by TPHA in some studies. Such rate was found 0.4% in the present study. Positivity rates of syphilis test vary between 0.29% and 7.5% in different countries of the world. The incidence of syphilis is more in Sub-Saharan regions than other regions of the world. However, different prevalence results are reported in aforementioned region. This is connected with methodological differences [16]. It was previously reported that *T*.*Pallidum* particle agglutination assay is more sensitive than rapid plasma regain and *T*.*Pallidum* hemaglutination assay [20].

African studies indicate a substantially decreasing trend in sero-prevalance of syphilis among blood donors in some countries [Cameroon, Tanzania, and Ghana]. Such decrease was about 70% and may be related to a positive effect of the prevention programs against HIV, as syphilis is a sexually transmitted disease. Moreover, the prevalence of syphilis detected in these African studies may have been increased by false-positive results due to other treponema species that are endemic in Africa, such as pian, bejel and pinta which cannot be differentiated from syphilis by serology tests [27]. In the present study, VDRL test positivity tended to decrease since 2014. Careful supervision during filling the donor inquiry form has been effective. Furthermore, it was considered that this may be associated with awareness rising of the community through information about safe blood donation.

The review on gender distribution in the present study revealed that VDRL positivity is significantly higher in female cases (0.5%) than males (0.3%) (p<0.05). The findings obtained in the study of Gureser et al. were similar to the present study. There was not any difference detected between the gender in the study conducted by Kader et al [5, 8]. Similarly, no difference was found between the genders in the study of Cao et al. from China [17].

There was significant difference in VDRL positivity rates according to the age range; review of the significance revealed that positivity rate for VDRL was significantly higher in the cases at 51 years of age and older when compared with the cases between 18 and 30 years of age as well as the cases at 31 and 40 years of age. The VDRL rates also increase by the increase in age. Kader et al. reported in their study that the cases with positive VDRL test were more in elder individuals. In the study of Noubiap from Cameron, positivity rates for syphilis increased after 40 years of age and were significantly higher over 50 years of age [14]. Cao et al. detected an increase in positive syphilis tests by increase in age [17].

In consideration of the trend in the present study by years, a significant difference is in HBsAg, anti-HCV positivity rates in 2014 when compared with other years. There was not any significant difference detected in HBsAg, anti-HCV positivity rates of other years. The difference between anti-HIV rates depending on the years was not significant. However, the difference in VDRL rates depending on the years was statistically significant. Such difference was compared with the increase detected in 2014; a significant difference is detected in HIV positivity rates of other years. Beyond relative high positive rates detected in the screening tests of year 2014, sero-prevalence rates appear to decrease within years (Fig 3).

Except some countries and centers, a decreasing trend is discussed in the positivity rates of the screening tests performed before blood transfusion in our country and globally. Such improvement in serological screening tests may be explained by the increase of awareness about the diseases spread by blood transfusion and increase in precautions taken to avoid such infections. Furthermore, it is reported that filling the investigation forms accurately and in detail provides elimination of 40% of HIV-infected cases as well as 1/4 of blood-borne infections [28].

Nucleic acid amplification test (NAT) was recently involved in blood banking as a specific test in many countries; these tests were started to be used for blood donor screening. However, high costs are the most important problem. Therefore, these tests are not used by every country and centre [8, 19]. It is not used as a routine screening test in our centre.

Nevertheless, despite all efforts and microbiological screening tests, there is a risk of infection with blood and blood products. In consideration of the window period, pre-seroconversion period of the infections as well as sensitivities of serological methods used, necessity of molecular methods (NAT) becomes forefront. Well-supported blood bank staff through periodical trainings, carefully filling the donor inquiry form and a careful physical examination by the physician, awareness increasing of the community about safe blood donation and generalization of vaccination programs would reduce the diseases that may spread through blood transfusion. A detailed filling of donor inquiry form for a safe donor selection would prevent the individuals at sero-negative period to be donor. In addition, accurate indication for blood use is still important to prevent unnecessary transfusion.

## Supporting information

S1 File DATA plos one Canan Eren_rates. (XLSX)

S2 File 1 min. data serology (+) HBsAg. (XLS)

S3 File 1 min. data serology (+) VDRL. (XLS)

S4 File 1 min. data. serology (+) anti.hcv. (XLS)

S5 File 1 Min. Data serology (+) Anti.HIV (1). (XLS)

## References

[1] Seitz R, Heiden M. Quality and safety in blood supply in 2010. Transfus Med Hemother 2010;37:112–7. 10.1159/000314497 20577599 PMC2889628

[2] Schweitzer A, Horn J, Mikolajczyk RT, Krause G, Ott JJ. Estimations of worldwide prevalence of chronic hepatitis B virus infection: a systematic review of data published between 1965 and 2013. Lancet 2015;386:1546–55. 10.1016/S0140-6736(15)61412-X 26231459

[3] World Health Organization. Global Health Observatory HIV/AIDS. http://www.who.int/gho/hiv/en. Accessed 20 May 2016.

[4] World Health Organization. Blood safety and availability. http://www.who.int/mediacentre/factssheets/fs279/en. Accessed 20 May 2016.

[5] Kader Ç, Erbay A, Biringel S, Gürbüz M. Seroprevalance of hepatitis B virus, hepatitis C virus, human immunodeficiency virus infections and syphilis in blood donors. Klimik J 2010;23(3):95–9.

[6] Uzun B, Güngör S, Demirci M. Seroprevalence of transfusion transmissible infections among blood donors in western part of Turkey: A six-year study. Transfusion and Apheresis Science 2013;49:511–5. 10.1016/j.transci.2013.02.039 23491864

[7] Glynn SA, Busch MP, Dodd RY, et al Emerging infectious agents and nation’s blood supply: responding to potential threats in the 21st century. Transfusion 2013;53:438–54. 10.1111/j.1537-2995.2012.03742.x 22690676 PMC3644861

[8] Güreser AS, Özçelik S, Boyacıoğlu Zİ, Özünel L, Yıldız Ü, Özkan AT. Seropositivity rates of HBsAg, anti-HCV, HIV and VDRL in blood donors in Corum, Turkey. Turk Hij Den Biyol Derg 2015; 72(2): 123–30.

[9] Akcam FZ, Uskun E, Avsar K, Songur Y. Hepatitis B virus and hepatitis C virus sero prevalence in rural areas of the western region of Turkey. Int J Infect Dis 2009;13:274–84. 10.1016/j.ijid.2008.07.005 18945630

[10] World Health Organization (WHO). Global hepatitis report 2017 Geneva: World Health Organization. P.2017.

[11] World Health Organization (WHO). Global Health Observatory (GHO) data HIV/AIDS. Available at http://www.who.int/gho/en./Accessed 11 April 2018.

[12] World Health Organization [WHO]. Report on global sexually transmitted infection surveillance 2015 Geneva: World Health Orginization. P. 2015.

[13] Temiz H, Gül K. The evaluation of HBsAg, anti-HIV and VDRL test results in blood donors. Turkish Journal of Infection 2008;22 [2]:79–82.

[14] Noubiap JJN, Joko WYA, Nansseu JRN, Tene UG, Siaka C. Sero-epidemiology of human immunodeficiency virus, hepatitis B and C viruses, and syphilis among first-time blood donors in Edea, Cameroon. International Journal of Infectious Diseases 2013;17:e832–e837. 10.1016/j.ijid.2012.12.007 23317526

[15] Tagny CT, Owusu-Ofori S, Mbanya D, Deneys V. The blood donor in sub-Saharan Africa: a review. Transfus Med 2010;20:1–10. 10.1111/j.1365-3148.2009.00958.x 19725906

[16] Okoroiwu HU, Okafor IM, Asemota EA, Okpokam DC. Seroprevalence of transfusion-transmissible infections [HBV, HCV, syphilis and HIV] among prospective blood donors in a tertiary health care facility in Calabar, Nigeria; an eleven years evaluation. BMC Public Health 2018;18:645 10.1186/s12889-018-5555-x 29788937 PMC5964952

[17] Cao WW, Zhou RR, Ou X, Shi LX, Xiao CQ, Chen TY, et al Prevalence of hepatitis B virus, hepatitis C virus, human immunodeficiency virus and Treponema pallidum infections in hospitalized patients before transfusion in Xiangya hospital Central South University, China from 2011 to 2016. BMC Infectious Diseases 2018;18[1]:145 10.1186/s12879-018-3051-7 29606088 PMC5879580

[18] Sheikh MY, Atla PR, Ameer A, Sadiq H, Sadler PC. Seroprevalance of Hepatitis B and C infections among healthy volunteer blood donors in the Central California Valley. Gut and Liver 2013;7[1]:66–73. 10.5009/gnl.2013.7.1.66 23423771 PMC3572322

[19] Chandekar SA, Amonkar GP, Desai HM, Valvi N, Puranik GV. Seroprevalance of transfusion transmitted infections in healthy blood donors: A 5-year tertiary care hospital experience. Journal of Laboratory Physicians 2017;9:4:283–87. 10.4103/0974-2727.214246 28966492 PMC5607759

[20] Tessema B, Yismaw G, Kassu A, Amsalu A, Mulu A, Emmrich F, et al Seroprevalance of HIV, HBV, HCV and syphilis infections among blood donors at Gondar University Teaching Hospital Northwest Ethiopia: declining trends over a period of five years. BMC Infect Dis 2010;10:111–7. 10.1186/1471-2334-10-111 20459703 PMC2881920

[21] Diarra A, Kouriba B, Baby M, Murphy E, Lefrere JJ. HIV, HBV, HCV and syphilis rate of positive donations among volunteer blood donors in Mali: lower rates among volunter blood donors. Transfus 2009:16(5–6):444–7. 10.1016/j.tracli.2009.09.004 19896404

[22] Turan H, Şerefhanoğlu K, Ünler GK, Arslan H. Seroprevalance of HBsAg, anti-HCV and their correlation to age and gender in blood donors in the province of Konya. Klimik J 2011;24[1]:36–9.

[23] Niazi SUK, Mahmood A, Alam M., Ghani E. Transfusion transmissible infections in blood donors from northern Pakistan: Three years experience (2010–2012). XIIth Annual conference of Asian association of transfusion medicine [AATM], 2–6 April 2016, Antalya/Turkey. Abstract book p368-369.

[24] İçel O, Köroğlu M, Demiray T, Özbayraktar S, Akel N, Gün R, et al Serotrends of the blood donor screening test results; Ten year review, Malatya. OTSBD 2016;1:3:1–7.

[25] Umolu PI, Okoror LE, Orhue P. Human immunodeficiency virus (HIV) seropositivity and hepatitis B surface antigenemia (HBsAg) among blood donors in Benin city, Edo state. Nigeria Afr Health Sci 2005;5[1]:55–8. 15843132 PMC1831904

[26] Ağuş N, Özkalay YN, Cengiz A, Şanal E, Sert H. HBsAg, anti-HCV, anti-HIV seroprevalence in blood donors. Ankem J 2008;22(1):7–9.

[27] Egglestone SI, Turner AJ. Serological diagnosis pf syphilis. PHLS syphilis serology working group. Commun Dis Public Health 2000;3:158–162. 11014025

[28] Van der Bij AK, Coutinho RA, Van der Poel CL. Surveillance of risk profiles among new repeat blood donors with transfusion-transmissible infections from 1995 through 2003 in Netherlands. Transfusion 2006;46(10):1729–36. 10.1111/j.1537-2995.2006.00964.x 17002629

